# An open-label, sequential, dose-finding study of peginesatide for the maintenance treatment of anemia in chronic hemodialysis patients

**DOI:** 10.1186/1471-2369-13-95

**Published:** 2012-08-30

**Authors:** Anatole Besarab, Steven N Zeig, Edouard R Martin, Pablo E Pergola, Frederick C Whittier, Raja I Zabaneh, Brigitte Schiller, Martha Mayo, Carol A Francisco, Krishna R Polu, Anne-Marie Duliege

**Affiliations:** 1 Henry Ford Hospital, Detroit, MI, USA; 2 Pines Clinical Research, Pembroke Pines, FL, USA; 3 South Florida Nephrology Associates, Lauderdale Lakes, FL, USA; 4 Renal Associates PA, San Antonio, TX, USA; 5 Clinical Research Ltd, Canton, OH, USA; 6 Northwest Louisiana Nephrology, Shreveport, LA, USA; 7 Satellite Healthcare, San Jose, CA, USA; 8 Affymax, Inc, Palo Alto, CA, USA

**Keywords:** Anemia, Chronic kidney disease, Erythropoiesis-stimulating agent, Epoetin alfa, Hemodialysis, Peginesatide

## Abstract

**Background:**

Peginesatide is a peptide-based erythropoiesis-stimulating agent that was designed and engineered to stimulate specifically the erythropoietin receptor dimer that governs erythropoiesis. The primary objective of this phase 2 dose-finding study was to determine the once-monthly peginesatide dosing strategy that would maintain hemoglobin within ±1.0 g/dL of baseline values after conversion from epoetin alfa; the safety of peginesatide was evaluated concurrently.

**Methods:**

Chronic hemodialysis patients on stable regimens of epoetin alfa were sequentially assigned to cohorts that differed on (1) how the peginesatide starting dose was determined (using a single epoetin alfa–to-peginesatide dose conversion ratio or a tiered, weight-based or absolute-dose conversion table) and on (2) whether or not a 1-week erythropoiesis-stimulating agent-free interval was used. Peginesatide doses were titrated to maintain hemoglobin levels within ±1.0 g/dL from baseline.

**Results:**

A total of 164 patients were enrolled and received intravenous peginesatide every 4 weeks for up to 6 doses; the duration of the study including follow-up was ≤29 weeks. Overall, the proportion of patients with hemoglobin levels within ±1.0 g/dL of baseline increased over the course of the study from 39% (Weeks 2–13) to 54% (Weeks 18–25). Cohorts that used tiered dose conversion tables trended towards having more stable peginesatide doses than did those cohorts that used a single dose conversion ratio. Moreover, cohorts that used an erythropoiesis-stimulating agent-free interval did not have the substantial initial increase in hemoglobin levels that was seen in those cohorts that did not use such an interval. In this study, the safety profile of peginesatide was consistent with those of marketed erythropoiesis-stimulating agents.

**Conclusions:**

The results of this study were used to guide the dosing regimens used subsequently in phase 3 studies. Once-monthly peginesatide is feasible in hemodialysis patients.

**Trial registration:**

ClinicalTrials.gov registration: NCT00228449

## Background

Anemia associated with chronic kidney disease (CKD) contributes to increased morbidity and a decreased quality of life [1,2]. Standard therapies for CKD-associated anemia are erythropoiesis-stimulating agents (ESAs), which have been shown to increase and maintain hemoglobin (Hb) levels leading to a decreased need for red blood cell transfusions [3]. These agents have also been associated with an increased risk of death and cardiovascular events in clinical trials targeting normalized hemoglobin levels [4-7].

Peginesatide, a synthetic ESA that was designed and engineered to stimulate specifically the erythropoietin receptor dimer that governs erythropoiesis, is composed of a dimeric peptide that is linked to a polyethylene glycol moiety [8]. The amino acid sequence of peginesatide is unrelated to that of erythropoietin; therefore, peginesatide is unlikely to induce a cross-reactive immune response against either endogenous or recombinant erythropoietin. Following intravenous administration, the half life of peginesatide is approximately 48 hours in dialysis patients and 25 hours in healthy subjects [7], suggesting that peginesatide clearance varies inversely with kidney function. Peginesatide has been shown to increase Hb in patients with anti-erythropoietin antibody-mediated pure red cell aplasia [9]. In healthy volunteers, peginesatide had a safety profile similar to that of placebo and was associated with a significant increase in Hb that sustained for over 1 month [10].

Results of a phase 2, dose-finding study of once-monthly (Q4W) peginesatide for the maintenance of Hb levels in chronic hemodialysis patients who were switched from epoetin alfa are reported herein. The primary objective of this study was to determine the peginesatide dosing strategy that would maintain Hb within ±1.0 g/dL of baseline values after conversion from epoetin alfa; the safety of peginesatide was also assessed.

## Methods

### Patients

Adult CKD patients were eligible if they had been clinically stable on hemodialysis for ≥6 months, had received epoetin alfa for ≥8 weeks, had 3 stable Hb values within 10.0 to 12.5 g/dL and evidence of adequate iron stores. The main exclusion criteria were intolerance to ESAs or parenteral iron supplementation, a history of antibodies to ESAs or pure red cell aplasia (see Additional file 1: Table S1 for complete inclusion and exclusion criteria).

### Study design

In this multicenter (14 centers in the United States), phase 2, open-label, sequential, dose-finding study, patients were administered intravenous Q4W peginesatide during dialysis, for a total of 6 doses. The duration of the study was ≤29 weeks. Participating sites received approval from their governing institutional review board (Henry Ford Health System IRB; Committee on Scientific Activities/IRB, Beth Israel Medical Center; Coast IRB, LLC; Human Subjects Research Committee, Minneapolis Medical Research Foundation). All patients provided written informed consent, and the study was performed in accordance with the Helsinki Declaration of 1975 (and as revised in 1983) and the ICH guidelines for Good Clinical Practice (ClinicalTrials.gov registration: NCT00228449).

The study design allowed for multiple cohorts of 15 patients each. Cohorts differed with respect to the strategy used to determine the starting dose of peginesatide: (1) either a single epoetin alfa–to-peginesatide dose conversion ratio or a tiered, weight-based or fixed-dose conversion table and (2) whether or not an ESA-free interval (epoetin alfa dosing stopped 1 week before the first dose) was used. In the first cohort, a single epoetin alfa–to-peginesatide dose conversion ratio of approximately 3000 U/wk of epoetin alfa to 1 mg/mo of peginesatide without an ESA-free interval was used. Subsequent cohorts were added on the basis of ongoing Hb and safety results, with 15 patients assigned sequentially to each cohort. Hemoglobin data from previous cohort(s) guided peginesatide starting doses and whether or not ESA-free intervals were used. The study allowed for repeating cohorts with identical dosing strategies.

After the first dose of peginesatide, each patient’s subsequent doses were adjusted using predetermined guidelines (Additional file 2: Table S2) to maintain Hb within ±1.0 g/dL of baseline; Hb levels, and adverse events (AEs) were assessed weekly. Investigators maintained iron levels according to the KDOQI treatment guidelines. Vital signs and clinical laboratory parameters were assessed periodically. 12-lead ECGs were evaluated during screening and 30 minutes after the first peginesatide dose. Blood samples to assess for antibodies to peginesatide were collected at each peginesatide dosing visit and at study termination. Patients were followed for ≥42 days after their last dose (unless they were enrolled in a long-term peginesatide extension study) or until the stabilization of AEs, whichever occurred later.

### Study drug

Peginesatide (formerly known as Hematide™; Affymax, Inc., Palo Alto, CA) was supplied as a preservative-free, aseptically manufactured, sterile parenteral solution provided in a 2-mL, single-use, clear glass vial. Each vial contained 1 mL of solution at a concentration of 10 mg/mL peginesatide in an acetate-buffered saline solution at pH 5.5 (±0.5) or in an isotonic phosphate-buffered solution at pH 6.0 (±0.5). The formulation was changed from an acetate-buffered solution to a phosphate-buffered solution to improve stability.

### Assessments

Dosing requirements were calculated using the median peginesatide dose for each cohort, stratified by the number of the injections. Efficacy variables for each cohort were mean Hb concentration and mean Hb change from baseline (the mean of the 3 most recent mid- or end-of-week predialysis Hb values collected in the 3 weeks before the start of the study). The number and percentage of patients who maintained Hb values within 9.5 to 13.0 g/dL and within 11.0 to 13.0 g/dL for each cohort were also assessed. The upper bound of 13.0 g/dL was used for these targets to permit Hb level variations within ±1.0 g/dL of baseline values (entry Hb levels could range from 10.0 to 12.5 g/dL); the upper bound of 12.5 g/dL was not used to avoid potentially excessive dose titration. Hemoglobin data collected subsequent to a phlebotomy, or 4 weeks subsequent to a transfusion or bleeding event, were excluded. Safety was assessed via the collection of AEs and SAEs assessed by the investigators as related or unrelated to the study drug. All AEs were graded by severity using the WHO toxicity criteria (ie, grades 1, 2, 3, and 4, considered mild, moderate, severe, and life threatening, respectively). Other safety end points were: reasons for study withdrawal, number of transfusions and phlebotomies, iron parameters, changes in clinical laboratory results, 12-lead ECGs and vital signs, and evaluations of antibodies to peginesatide (both neutralizing and non-neutralizing).

### Statistical analysis

Cohorts with identical starting doses and dosing strategies were summarized together. Number and percentage of responses in each category were used for discrete measures, and the number of observations, mean, median, and standard deviation were used for continuous measures. Summary statistics were presented for each dose group. Patients who received ≥1 dose of study drug were included in all analyses. Because of the small numbers of patients in each of the cohorts and their sequential nature, no formal comparisons, including baseline characteristics, between cohorts were performed.

## Results

### Patient characteristics and disposition

**Figure 1:**
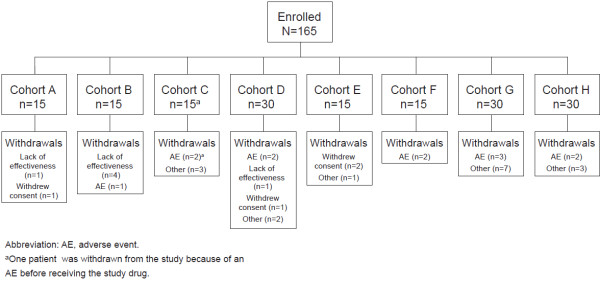
Patient disposition.

This study was conducted from July 2005 to May 2007. A total of 165 patients were enrolled (Figure 1) and assigned to 1 of 11 cohorts of 15 patients each; 3 different pairs of cohorts had identical starting doses and dosing strategies and were combined into single cohorts of 30 patients each (cohorts D, G, and H; Table 1), leaving 8 cohorts for analysis (Table 1). Single epoetin alfa–to-peginesatide dose conversion ratios were used in Cohorts A to E, whereas tiered (weight-based or absolute-dose) dose conversion tables were used in Cohorts F to H. Conversion from epoetin alfa to peginesatide without an ESA-free interval was investigated in Cohorts A, B, C, G, and H, whereas conversion with an ESA-free interval was investigated in Cohorts D, E, and F.

**Table 1:** Dosing cohorts

**Cohort**	**n**	**Conversion Strategy**	**Weekly Epoetin Alfa Dose**	**Peginesatide Starting Dose**	**Epoetin Alfa–to-Peginesatide Dose Conversion Ratio (U/kg/wk:mg/kg/mo)**	**1-Week ESA-Free Interval**
A	15	Dose conversion ratio	NA	NA	3030:1	No
B	15	Dose conversion ratio	NA	NA	2439:1	No
C	14	Dose conversion ratio	NA	NA	2000:1	No
D	30	Dose conversion ratio	NA	NA	2000:1	Yes
E	15	Dose conversion ratio	NA	NA	1515:1	Yes
F	15	Tiered weight-based	<100 U/kg	0.050 mg/kg	<2000:1	Yes
			100 to 150 U/kg	0.075 mg/kg	1333 to 2000:1	
			> 150 to 200 U/kg	0.100 mg/kg	1500 to 2000:1	
			>200 U/kg	0.150 mg/kg	>1333:1	
G	30	Tiered weight-based	<100 U/kg	0.050 mg/kg	<2000:1	No
			100 to 150 U/kg	0.075 mg/kg	1333 to 2000:1	
			>150 to 200 U/kg	0.100 mg/kg	1500 to 2000:1	
			>200 U/kg	0.150 mg/kg	>1333:1	
						
H	30	Tiered fixed-dose	<8000 U	4.0 mg	<2000:1	No
			8000 to 16000 U	6.0 mg	1333 to 2666:1	
			>24000 U	12.0 mg	1333 to 2000:1	
				16.0 mg	>1500:1	

Abbreviation: ESA, erythropoiesis-stimulating agent.

All but 1 of the 165 enrolled patients received ≥1 dose of peginesatide and were included in the analyses. One patient (enrolled in Cohort C), who experienced a fatal cardiac arrest before receiving the study drug, was not included.

Thirty-eight patients were withdrawn from the study: 12 patients because of AEs (detailed in the Safety section), 4 patients withdrew consent, and 16 patients for other reasons including the use of prohibited medication (epoetin alfa, n = 4; darbepoetin alfa, n = 2; cocaine, n = 1) and patient moved geographically or transferred to another non-participating center (n = 3). Six patients were withdrawn because of a lack of effectiveness; 5 were in cohorts that had the lowest relative starting doses of peginesatide (Cohorts A and B) and 1 was in a cohort that had a higher relative starting dose (Cohort D). These 6 patients received 3 or 4 doses before discontinuation, and their mean change from baseline Hb level to the time of discontinuation was -1.1 g/dL.

Baseline and demographic characteristics are detailed in Table 2. The majority of patients were men (57%) and black (56%). The median dose of epoetin alfa at baseline was 137 U/kg/wk and the mean (SD) Hb at baseline was 11.5 (0.6) g/dL. During the 8 weeks before study drug treatment, 141 patients (86%) received intravenous epoetin alfa, 22 patients (13%) received subcutaneous epoetin alfa, and 1 patient (<1%) received both intravenous and subcutaneous epoetin alfa.

**Table 2:** Patient baseline and demographic characteristics

**Characteristic**	**Cohort A n = 15**	**Cohort B n = 15**	**Cohort C n = 14**	**Cohort D n = 30**	**Cohort E n = 15**	**Cohort F n = 15**	**Cohort G n = 30**	**Cohort H n = 30**	**Total (N = 164)**
Age, mean (SD), y	62.8 (18.8)	59.7 (14.2)	55.9 (14.8)	59.1 (14.3)	65.7 (11.8)	54.4 (18.3)	57.1 (16.0)	59.3 (13.8)	59.1 (15.2)
Men, no. (%)	6 (40.0)	9 (60.0)	10 (71.4)	17 (56.7)	8 (53.3)	10 (66.7)	13 (43.3)	21 (70.0)	94 (57.3)
Black, no. (%)	5 (33.3)	10 (66.7)	7 (50.0)	15 (50.0)	9 (60.0)	10 (66.7)	18 (60.0)	18 (60.0)	92 (56.1)
Hb, mean (SD), g/dL	11.6 (0.6)	11.6 (0.4)	11.7 (0.6)	11.5 (0.7)	11.4 (0.6)	11.5 (0.5)	11.5 (0.6)	11.5 (0.7)	11.5 (0.6)
Kt/V, mean (SD)	1.87 (0.83)	1.61 (0.18)	1.52 (0.22)	1.59 (0.27)	1.69 (0.24)	1.61 (0.29)	1.79 (0.33)	1.69 (0.24)	1.68 (0.36)
Ferritin, mean (SD), ng/mL	724.1 (212.2)	633.2 (321.2)	635.6 (423.2)	789.2 (510.0)	887.7 (413.4)	1073.9 (643.4)	665.6 (456.6)	564.0 (401.4)	727.1 (458.9)
TSAT, mean (SD),%	36.4 (9.5)	32.5 (4.9)	43.1 (12.0)	50.4 (19.4)	52.8 (17.1)	57.6 (16.3)	31.6 (10.3)	33.3 (12.0)	41.2 (16.5)
CHr, mean (SD), pg	31.7 (1.9)	32.1 (2.6)	32.2 (2.8)	32.4 (2.0)	33.2 (1.7)	32.3 (2.4)	32.3 (1.9)	32.7 (2.3)	32.4 (2.2)
Epoetin alfa dose, median	143	130	161	124	109	118	132	160	137
(range)	(92–377)	(70–288)	(67–365)	(63–277)	(59–214)	(72–231)	(65–327)	(80–367)	(59–377)
U/kg/wk	9997	12444	12598	9603	9547	7600	7398	7598	8567
U/wk	(6371–33650)	(4634–21709)	(6593–36590)	(3602–24597)	(4544–30445)	(5066–10140)	(4797–10666)	(965–14820)	(965–36590)
Primary cause of CKD, no. (%)									
Diabetes	6 (40.0)	6 (40.0)	6 (42.9)	12 (40.0)	6 (40.0)	3 (20.0)	11 (36.7)	17 (56.7)	67 (40.9)
Hypertension	4 (26.7)	4 (26.7)	2 (14.3)	13 (43.3)	6 (40.0)	9 (60.0)	15 (50.0)	8 (26.7)	61 (37.2)
Other	5 (33.3)	5 (33.3)	6 (42.9)	5 (16.7)	3 (20.0)	3 (20.0)	4 (13.3)	5 (16.7)	36 (22.0)

Abbreviations: SD, standard deviation; Hb, hemoglobin; Kt/V, urea clearance per volume; TSAT, transferrin saturation; CHr, reticulocyte hemoglobin content; CKD, chronic kidney disease.

### Peginesatide dosing

**Figure 2:**
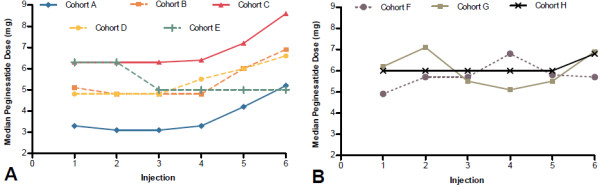
Effects of dose conversion ratios (A) vs tiered conversion tables (B) on median peginesatide doses over time.

Overall, the median peginesatide dose increased from Dose 1 (0.073 mg/kg) to Dose 6 (0.085 mg/kg). The median dose of peginesatide tended to increase more in those cohorts in which a single epoetin alfa–to-peginesatide dose conversion ratio was used (Figure 2A), except for Cohort E, in which the highest relative starting dose (mg/kg) of peginesatide was used. Although median peginesatide doses also tended to increase in those cohorts in which tiered conversion tables were used (Figure 2B), this effect was not as pronounced as it was in those cohorts in which single dose conversion ratios were used. Of note, peginesatide doses were adjusted after the first dose to maintain Hb within ±1 g/dL of the baseline concentration and comparisons of dose requirements between groups is complicated by differences in the initial dosing regimen.

### Efficacy

**Figure 3:**
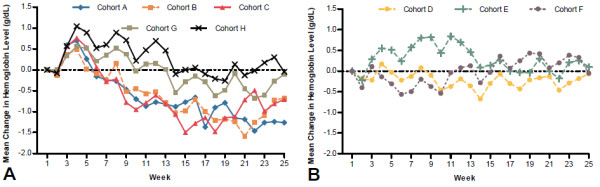
Effects of not using (A) vs using (B) an erythropoiesis-stimulating agent–free interval on mean hemoglobin levels over time.

Overall, the proportion of patients with Hb levels within ±1.0 g/dL of baseline increased over the course of the study from 39% (Weeks 2–13) to 54% (Weeks 18–25). Following the initial dose, increases in mean Hb levels during the first several weeks were evident in those cohorts that did not use an ESA-free interval (Cohorts A, B, C, G, and H; Figure 3A). This initial increase generally was not evident in those cohorts using an ESA-free interval (Cohorts D, E, and F; Figure 3B); the only exception to this was Cohort E. This may reflect the higher relative starting dose that was used in Cohort E, compared with that of Cohort D. In addition, a single epoetin alfa–to-peginesatide dose conversion ratio was used in Cohort E, whereas a tiered weight-based dose conversion table was used in Cohort F.

Hemoglobin levels were maintained within 9.5 to 13.0 g/dL for most patients during Weeks 2 to 13 (75%) and Weeks 14 to 25 (79%), regardless of cohort (Table 3); Hb levels were maintained within 11.0 to 13.0 g/dL in fewer patients (Additional file 3: Table S3). The lower proportion of patients may reflect that the dose adjustment guidelines were designed to keep Hb within ±1.0 g/dL of baseline and did not target a Hb concentration between 11.0 and 13.0 g/dL. Overall, however, Hb levels were maintained within 9.5 to 13.0 g/dL and within 11.0 to 13.0 g/dL in a higher proportion of patients in those cohorts in which an ESA-free interval was used than in those cohorts in which an ESA-free interval was not used (Tables 3 and Additional file 3: Table S3).

**Table 3:** Patients with hemoglobin concentrations within 9.5 to 13.0 g/dL

**Interval**	**Cohort A**	**Cohort B**	**Cohort C**	**Cohort D**	**Cohort E**	**Cohort F**	**Cohort G**	**Cohort H**	**Total l**	**Cohorts With an ESA- Free Interval (D, E, F)**	**Cohorts Without an ESA- Free Interval (A, B, C, G, H)**
Weeks 2–13, n	14	15	12	25	15	13	25	29	148	53	95
no. (%)	9 (64.3)	12 (80.0)	9 (75.0)	21 (84.0)	11 (73.3)	11 (84.6)	20 (80.0)	18 (62.1)	111 (75.0)	43 (81.1)	68 (71.6)
Weeks 14-25, n	6	10	10	25	13	13	24	26	127	51	76
no. (%)	5 (83.3)	9 (90.0)	6 (60.0)	21 (84.0)	12 (92.3)	10 (76.9)	21 (87.5)	16 (61.5)	100 (78.7)	43 (84.3)	57 (75.0)

First row of each set indicates the total number of patients, second row, the number within range and the percentage.

### Safety

Adverse events were reported in 137 of 164 patients (84%), of which, 93 patients (68%) had AEs considered mild to moderate in severity. Table 4 details the most common (≥5%) AEs. The most common (>1%) AEs considered by the investigators to be possibly related to peginesatide were fatigue (1.8%), decreased Hb (1.8%), asthenia (1.2%), and rash (1.2%). Ten out of 164 patients reported arteriovenous graft complications; a total of 12 patients had access-related complications. The Hb levels near the time of the complications were between 7.9 and 13 g/dL in all but one patient, who had a Hb of 14.7 g/dL.

**Table 4:** Most frequently (occurring in ≥5% of patients) reported adverse events

**Treatment-Emergent Adverse Event, no. (%)**	**Total n = 164**
Upper respiratory tract infection	19 (11.6)
Diarrhea	16 (9.8)
Nausea	15 (9.1)
Vomiting	15 (9.1)
Cough	13 (7.9)
Dyspnea	13 (7.9)
Muscle spasms	13 (7.9)
Pyrexia	13 (7.9)
Headache	12 (7.3)
Asthenia	11 (6.7)
Fatigue	11 (6.7)
Back pain	10 (6.1)
Hypertension	10 (6.1)
Pain in extremity	10 (6.1)
Hypocalcemia	9 (5.5)
Hypotension	9 (5.5)

Serious adverse events were reported in 47 of 164 patients (29%). Table 5 details the most common (>1 patient) serious adverse events (SAEs). A single SAE (infusion-related reaction, described below) was considered possibly related to peginesatide. The incidence of AEs ranged from 60% to 93% and SAEs ranged from 13% to 40% across cohorts without evidence of a relationship between starting dose and the incidence of either AEs or SAEs.

**Table 5:** Most frequently (occurring in >1 patient) reported serious adverse events

**Serious Adverse Event, no. (%)**	**Total n = 164**
Congestive cardiac failure	6 (3.7)
Pneumonia	4 (2.4)
Acute myocardial infarction	3 (1.8)
Convulsion	3 (1.8)
Pulmonary edema	3 (1.8)
Sepsis	3 (1.8)
Catheter sepsis	2 (1.2)
Coronary artery disease	2 (1.2)
Hypotension	2 (1.2)
Pneumonia, aspiration	2 (1.2)
Pulmonary embolism	2 (1.2)
Respiratory failure	2 (1.2)

Five deaths occurred during the course of the study, 1 of which occurred before dosing. The four deaths that occurred after administration of the first dose of study drug were due to pneumonia and sepsis, cardiac arrest, cardiorespiratory arrest, and sepsis and respiratory failure. None of the deaths were reported as being related to peginesatide and all were typical of the end stage renal disease population studied.

Twelve patients were withdrawn from the study because of AEs. Four of these patients died (as noted above). Five patients were withdrawn because of serious, nonfatal events of study drug infusion-related reaction, aspiration pneumonia and myocardial infarction, leukocytoclastic vasculitis, aspiration, and vascular pseudoaneurysm. The event of infusion-related reaction (ie, pruritus, anxiety, nausea, hypotension, and vomiting) began 2 minutes after the patient’s first peginesatide dose. After receiving intravenous (IV) fluids and antihistamine treatment, the patient recovered and was discharged on the same day with no further symptoms; peginesatide treatment was discontinued. The event of leukocytoclastic vasculitis began 35 days after the patient’s second peginesatide dose (11.2 mg), 4 weeks after beginning clopidogrel treatment, and 11 days after receiving a flu vaccine. The investigator reported the event as unlikely to have been related to study drug. Two patients were withdrawn after receiving renal transplants. One patient was withdrawn after developing a rash that was initially pruritic in nature. Of the AEs that led to patient withdrawal from the study, only the events of rash/pruritic rash and infusion-related reaction were considered by the investigators to be possibly related to peginesatide administration.

A total of 8 patients (4.9%) received red blood cell transfusions (Cohort D, n = 4; Cohort F, n = 1; Cohort G, n = 2; Cohort H, n = 1). Five patients received transfusions exclusively for the treatment of anemia. Of the remaining 3 patients, 1 received two transfusions (1 for hemorrhagic shock and 1 for anemia); 1 received multiple transfusions for shock, multiple trauma (fractures), as a postsurgical procedure, and for anemia; and 1 was transfused for postoperative hemorrhage.

One patient in Cohort H had a high Hb concentration approximately 1 week after receiving the fifth peginesatide injection that remained elevated during the next 11 weeks (range, 12.4 g/dL to 16.7 g/dL). The patient was phlebotomized (500 mL) 91 days after Dose 5 (201 days after Dose 1) with a decline in Hb level from 16.7 g/dL the day before the phlebotomy to 13.4 g/dL the day after the procedure and 11.8 g/dL one week after. This patient was withdrawn from the study because the need for phlebotomy was a withdrawal criterion.

Iron levels were satisfactorily maintained throughout the study, with mean baseline and end-of-study ferritin values of 727 ng/mL and 742 ng/mL, respectively; mean baseline and end-of-study transferrin saturation (TSAT) values of 41% and 49%, respectively; and mean baseline and end-of-study reticulocyte Hb contents of 32.4 pg and 34.2 pg, respectively.

Five patients had elevations in alanine aminotransferase (ALT) ≥3x upper limit of normal (ULN) and/or aspartate aminotransferase (AST) ≥3x ULN. Three patients had transient elevations in ALT and/or AST; the other 2 patients had elevated values at baseline which were sustained during the study. One patient had transient elevation in total bilirubin ≥2x ULN. All but 1 of these patients were receiving concomitant heparin, statin, and/or aspirin therapy, which have been associated with elevations in liver function tests. None of the ALT or AST elevations were associated with a concurrent rise in total bilirubin ≥2x ULN. No patients developed antibodies specific to peginesatide.

Mean systolic and diastolic blood pressure fluctuated during the trial for each cohort, with values similar to those recorded during the screening period while subjects were receiving epoetin alfa.

In general, there were no changes in the interpretation of ECG results at baseline and 30 minutes after the first peginesatide dose. No patient had an ECG abnormality interpreted by the investigator as clinically significant that was not present at screening.

## Discussion

The primary purpose of this study was to determine the optimal peginesatide dosing strategy to maintain Hb levels in hemodialysis patients who were previously treated with epoetin alfa. The strategies that were investigated were 1) single epoetin alfa–to-peginesatide dose conversion ratios versus tiered conversion tables; and 2) whether or not an ESA-free interval was used. Overall, 53.6% of patients in this study achieved a Hb level within ±1.0 g/dL of their baseline level by the end of the study (ie, Weeks 18–25). Of note, per-protocol peginesatide dose adjustments were allowed after the first dose.

An increase in the median peginesatide dose requirement was observed in cohorts that used a single epoetin alfa–to-peginesatide dose conversion ratio, whereas this general trend was attenuated in cohorts that used tiered conversion tables. Initial peginesatide doses that resulted from a single dose conversion ratio may have been inadequate for patients with high epoetin alfa dose requirements, indicating that interpatient variability in dose requirements appears to have been better addressed by tiered dose conversion tables than it was by a single dose conversion ratio. These results also suggest that epoetin alfa–to-peginesatide dose conversion may not be linear. An alternative explanation is that the initial peginesatide doses that resulted from tiered dose conversion tables were higher than those that resulted from a single dose conversion ratio. However, Cohort C (single dose conversion ratio) had a similar starting dose as did Cohorts G and H (tiered dose conversion tables), and the median dose for Cohort C increased substantially during the course of the study, whereas dose increases in Cohorts G and H were less pronounced. These results suggest that the differences in dose requirements between dosing strategies were not solely due to higher initial doses.

Mean Hb values tended to increase during the first several weeks after initiating peginesatide treatment in those cohorts that did not use an ESA-free interval. Moreover, Hb levels were maintained within 9.5 to 13.0 g/dL and within 11.0 to 13.0 g/dL in a higher proportion of patients in those cohorts in which an ESA-free interval was used. These data indicate that an ESA-free interval appears to minimize the initial Hb rise after conversion and may be beneficial when switching patients from epoetin alfa to peginesatide. A potential explanation for this may be an additive effect of the two agents. Epoetin alfa-induced reticulocytosis (the pharmacodynamic effect) peaks approximately 1 to 2 weeks after administration [11], substantially beyond its thrice-weekly dosing schedule. This effect could have added to the pharmacodynamic effect of peginesatide on erythropoiesis and produced an initial increase in Hb levels from baseline, an effect not seen when the start of peginesatide was delayed by 1 week.

Similarly to Cohorts A, B, and C, Cohorts G and H did not have a 1-week ESA-free interval, and an increase in Hb levels was evident in the first weeks after conversion to peginesatide; however, Hb levels returned to near-baseline in Cohorts G and H, whereas they declined to below baseline levels in Cohorts A, B, and C. This may be explained by the use of tiered conversion tables in Cohorts G and H versus the use of a single dose conversion ratio in Cohorts A, B, and C.

The results of this study did not raise any new concerns regarding the safety profile of peginesatide; that is, no unexpected trends emerged with regard to the severity or incidence of AEs, and the safety profile of peginesatide appeared to be consistent with those of marketed ESAs [12]. Most of the reported AEs were considered to be unrelated to peginesatide and were generally mild or moderate in intensity. Fatigue, decreased Hb levels, asthenia, and rash were the most common AEs (>1%) considered by the investigators to be possibly related to peginesatide. The observed SAEs were consistent with events that have been described in dialysis populations with multiple comorbidities [13] and the incidence of SAEs was similar to that reported in a dose-finding study of peginesatide in CKD non-dialysis patients [14]. One SAE (infusion-related reaction) was considered possibly peginesatide related. No neutralizing or non-neutralizing antibodies against peginesatide were detected in any patient. Five deaths occurred during the course of the study, 1 of which occurred before dosing. Lack of effect was the reason for study discontinuation in less than 5% of patients. This lack of effect could be explained by transient hyporesponse, which lasts several months and can develop in previously stable patients, or suboptimal dose conversion. This aspect is better studied in a randomized controlled trial with an active comparator.

This study had several limitations. For example, it was open label and did not have an active control arm. Further, the proportion of Black patients was high (56%) relative to the proportion in the general US population (37%) [15]. Black patients tend to have higher ESA dose requirements than do patients of other races [16], complicating extrapolation of the average dose requirements of the general population from results of the current study. However, the proportion of Black patients was similar across cohorts (other than a somewhat lower proportion in Cohort A), attenuating the possibility that race had an effect on cross-cohort comparisons. Ferritin and TSAT levels were somewhat high overall at baseline with mean values greater than the minimum requirements for entry (TSAT >20%, ferritin >200 ng/mL). However, data from the Centers for Medicare and Medicaid Services Clinical Performance Measures project indicated that mean values for both TSAT and ferritin were progressively increasing in the US during the period that the current study was conducted [17]. Although there were some substantial differences in these parameters between cohorts, which may affect interpretation of the dosing results, examination of the mean values for TSAT and Hb levels across cohorts suggest that adequate iron delivery to the erythron was likely during the study. Additionally, patients entered the trial with Hb values between 10.0 and 12.5 g/dL and doses were titrated to maintain Hb within ±1.0 g/dL of their baseline value. However, since the time of the conduct of this study, clinical practice guidelines and updates to prescribing information have narrowed the Hb target range to less than 11.0 g/dL in the US and between 10.0 and 12.0 g/dL in the EU to minimize risks associated with normalizing Hb levels to 13.0 g/dL. Therefore, extrapolating dose requirements from this study are limited by these differences. Phase 3 randomized, active controlled studies with peginesatide have since been conducted and will help to inform the assessment of dosing, efficacy, and safety in the dialysis patient population [18-20].

## Conclusions

The purpose of this study was to evaluate the use of dose conversion ratios and tiered dose conversion with and without an ESA-free period as dosing strategies for peginesatide. The results showed that increases in peginesatide dose requirements were less apparent in cohorts in which tiered conversion tables were used and Hb levels appeared more stable in those cohorts in which an ESA-free interval was used. These results guided dosing strategies for randomized, controlled phase 3 peginesatide trials in which patients were randomized to continued epoetin treatment or conversion to peginesatide treatment. Preliminary results of the phase 3 trials have been reported in abstract form [18-20].

## Competing interests

Anatole Besarab, MD is a consultant for Affymax, Inc., Akebia, Amgen, Inc., Bayer AG, FibroGen, Inc., Hoffmann-La Roche Ltd, and Rockwell Medical; is on the Speakers Bureau for Affymax, Inc., Takeda Pharmaceuticals USA, Inc., Amgen, Inc., Hoffmann-La Roche Ltd, and Sanofi US; and has received grants from Affymax, Inc., Akebia Therapeutics, Inc., Amgen, Inc., FibroGen, Inc., Rockwell Medical, REATA Pharmaceuticals, Inc., Keryx Biopharmaceuticals, Inc., and Shire.

Steven Zeig is a consultant for Affymax, Inc. and Amgen, Inc.; is a member of the Scientific Advisory Board for Amgen, Inc., and is a Study Investigator for Affymax Inc., Amgen, Inc., Takeda Pharmaceuticals USA, Inc., Merck & Co., Inc., Keryx Biopharmaceuticals, Inc, Forest Research Laboratories, and Fibrogen, Inc.

Frederick Whittier, MD is a Study Investigator for Affymax, Inc., Keryx Biopharmaceuticals, Inc., Bristol-Meyers Squibb Company, Astra Zeneca, and Novo Nordisk A/S.

Edouard Martin, MD is a Study Investigator for Affymax, Inc.

Raja Zabaneh, MD is a Study Investigator and received research funding from Affymax, Inc.

Pablo Pergola, MD PhD has received research support from Affymax, Inc.

Brigitte Schiller, MD is a member of the Scientific Advisory Boards for Affymax, Inc. and NxStage Medical, Inc.

Anne-Marie Duliege, MD, MS; Carol Francisco, PhD; Martha Mayo, PharmD; Krishna R. Polu, MD are employees of Affymax, Inc.

## Authors' contributions

CAF, AMD, and MM were involved in the design of the study as well as the analysis and interpretation of the data. KRP was involved in the analysis and interpretation of data for this study. AB, SNZ, ERM, PEP, FCW, RIZ, and BS were investigators for this study. All authors were involved in writing the manuscript and revising it critically for important intellectual content. All authors approved the final manuscript.

## Pre-publication history

The pre-publication history for this paper can be accessed here:

http://www.biomedcentral.com/1471-2369/13/95/prepub

## Supplementary Material

Additional file 1 **Table S1.** Complete inclusion and exclusion criteria. Click here for file

Additional file 2 **Table S2.** Guidelines for dose adjustments and phlebotomies. Click here for file

Additional file 3 **Table S3.** Patients with hemoglobin concentrations within 11.0 to 13.0 g/dL. Click here for file
