# Supplementary material for: An open-label, sequential, dose-finding study of peginesatide for the maintenance treatment of anemia in chronic hemodialysis patients
Source: BMC Nephrol. 2012 Aug 30;13:95. doi: 10.1186/1471-2369-13-95 (PMC3511162; doi:10.1186/1471-2369-13-95)
Supplement: Additional file 1 — Table S1. Complete inclusion and exclusion criteria. [file 1471-2369-13-95-S1.doc]

**Supplementary Table 1. Complete Inclusion and Exclusion Criteria.**

| **Inclusion Criteria** |
| --- |
| Patient was informed of the investigational nature of this study and gave written, witnessed informed consent in accordance with institutional, local, and national guidelines |
| Males or females ≥18 years of age. Premenopausal females (with the exception of those who were surgically sterile) must have had a negative pregnancy test at screening; those who were sexually active must have practiced a highly effective method of birth control for at least 4 weeks prior to study start, and have been willing to continue contraception until at least 4 weeks after the last dose of study drug. A highly effective method of birth control was defined as one that results in a low failure rate (ie, less than 1% per year) when used consistently and correctly, such as implants, injectables, combined oral contraceptives, some intrauterine devices, sexual abstinence (only acceptable if practiced as a lifestyle), or vasectomized partner |
| Clinically stable on hemodialysis for ≥6 months prior to study start |
| Urea clearance/volume (Kt/V) ≥1.2 within the 4 weeks prior to study start |
| Epoetin alfa maintenance therapy ≥60 and ≤375 U/kg/week continuously prescribed for 8 weeks prior to study start. In the last 3 weeks prior to study start, variation in prescribed total weekly dose must have been ≤25% from the mean of the last three prescribed total weekly doses |
| Three mid- or end-of-week Hb values ≥10.0 and ≤12.5 g/dL in the 3 weeks prior to study start with ≤1.2 g/dL difference between the three values |
| One serum ferritin level ≥100 μg/L, or one TSAT ≥20%, or one CHr ≥29 pg within 4 weeks prior to study start |
| One serum folate level above the LLN during the 4 weeks prior to study start |
| One vitamin B_12_ level above the LLN during the 4 weeks prior to study start |
| Weight ≥45 kg within the 4 weeks prior to study start |
| One white blood cell count ≥3.0 x 10^9^/L within 4 weeks prior to study start |
| One platelet count ≥100 x 10^9^/L and ≤500 x 10^9^/L within 4 weeks prior to study start |

| **Exclusion Criteria** |
| --- |
| Known intolerance to ESAs |
| History of antibodies to ESAs or history of pure red cell aplasia |
| Known intolerance to parenteral iron supplementation |
| RBC transfusion within 12 weeks prior to study start |
| Hemoglobinopathy (eg, homozygous sickle-cell disease, thalassemia of all types) |
| Known hemolysis |
| Chronic, uncontrolled, or symptomatic inflammatory disease (eg, rheumatoid arthritis, systemic lupus erythematosus) |
| C-reactive protein >30 mg/L within the 4 weeks prior to study start |
| Moderate or significant infection within 2 weeks prior to study start |
| Known coagulation disorder based on clinical context and laboratory (activated partial thromboplastin time or international normalized ratio results) |
| Temporary (untunneled) dialysis access catheter |
| Uncontrolled or symptomatic secondary hyperparathyroidism |
| Poorly controlled hypertension within 4 weeks prior to study start, according to the Investigator’s clinical judgment (eg, systolic ≥170 mmHg or diastolic ≥100 mmHg on repeat measurements) |
| Any history of multiple significant drug allergies |
| History of severe or unstable reactive airway disease within the previous 10 years |
| Epileptic seizure in the 6 months prior to screening |
| Chronic congestive heart failure (New York Heart Association Class IV) |
| High likelihood of early withdrawal or interruption of the study (e.g., myocardial infarction; severe or unstable coronary artery disease; stroke; respiratory, autoimmune, neuropsychiatric, or neurological abnormalities; liver disease including active hepatitis B or C; active human immunodeficiency virus [HIV]; or any other clinically significant medical diseases or conditions in the prior 6 months that may, in the Investigator’s opinion, interfere with safety, assessment, or follow-up of the patient) |
| Evidence of malignancy within the past 5 years (except for nonmelanoma skin cancer) |
| Life expectancy <12 months |
| Anticipated elective surgery during the study period, which may be expected to lead to significant blood loss, including vascular access surgery (such as an arteriovenous fistula or graft) expected within 12 weeks after first study drug administration |
| Previous exposure to any investigational agent within 6 weeks before study start or planned receipt during the study period |

Abbreviations: Hb, hemoglobin; TSAT, transferrin saturation; CHr, reticulocyte hemoglobin content; ESAs, erythropoiesis-stimulating agents; LLN, lower limit of normal; RBC, red blood cell.
