# Supplementary material for: An open-label, sequential, dose-finding study of peginesatide for the maintenance treatment of anemia in chronic hemodialysis patients
Source: BMC Nephrol. 2012 Aug 30;13:95. doi: 10.1186/1471-2369-13-95 (PMC3511162; doi:10.1186/1471-2369-13-95)
Supplement: Additional file 2 — Table S2. Guidelines for dose adjustments and phlebotomies. [file 1471-2369-13-95-S2.doc]

Supplementary Table 2. Guidelines for Dose Adjustments and Phlebotomies.

| **Hemoglobin Parameter Prior to Dosing** | **Action** |
| --- | --- |
| ≥0.5 g/dL below baseline | 25% dose increase |
| ≥1.0 g/dL below baseline and <10.5 g/dL^a^ | 50% dose increase |
| 13.0–13.4 g/dL^b^ | 25% dose decrease |
| >1.0 g/dL above baseline^c^ | 25% dose decrease |
| ≥13.5 g/dL^b,d^ | Dose delay until Hb was <13.5 g/dL, and the dose was decreased by 25% |
| ≥14.0 and <16.0 g/dL^e^ | Patient was phlebotomized at the discretion of the investigator |
| ≥16.0 g/dL^e^ | Patient was phlebotomized |

Abbreviation: Hb, hemoglobin.

^a^ This was added as an amendment to the original protocol.

^b^ The Hb level must have been confirmed (2 consecutive values) within the 2-week period before dosing.

^c^ The Hb level must have been confirmed within any 2-week period.

^d^ During the course of the study, this criterion was changed from >12.5 g/dL; if this occurred, the patient’s dose was delayed until the Hb value was <12.0 g/dL, after which time peginesatide was restarted at a 25% lower dose.

^e^ The Hb increase must have been confirmed.
