# Supplementary material for: An open-label, sequential, dose-finding study of peginesatide for the maintenance treatment of anemia in chronic hemodialysis patients
Source: BMC Nephrol. 2012 Aug 30;13:95. doi: 10.1186/1471-2369-13-95 (PMC3511162; doi:10.1186/1471-2369-13-95)
Supplement: Additional file 3 — Table S3. Patients with hemoglobin concentrations within 11.0 to 13.0 g/dL. [file 1471-2369-13-95-S3.doc]

**Supplementary Table 3. Patients With Hemoglobin Concentrations Within 11.0 to 13.0 g/dL.**

| **Interval** | **Cohort A** | **Cohort B** | **Cohort C** | **Cohort D** | **Cohort E** | **Cohort F** | **Cohort G** | **Cohort H** | **Total** | **Cohorts With an ESA-Free Interval (D, E, F)** | **Cohorts Without an ESA-Free Interval (A, B, C, G, H)** |
| --- | --- | --- | --- | --- | --- | --- | --- | --- | --- | --- | --- |
| Weeks 2-13, n | 14 | 15 | 12 | 25 | 15 | 13 | 25 | 29 | 148 | 53 | 95 |
| no. (%) | 3 (21.4) | 5 (33.3) | 4 (33.3) | 14 (56.0) | 7 (46.7) | 5 (38.5) | 9 (36.0) | 9 (31.0) | 56 (37.8) | 26 (49.1) | 30 (31.6) |
| Weeks 14-25, n | 6 | 10 | 10 | 25 | 13 | 13 | 24 | 26 | 127 | 51 | 76 |
| no. (%) | 1 (16.7) | 1 (10.0) | 1 (10.0) | 11 (44.0) | 6 (46.2) | 5 (38.5) | 8 (33.3) | 8 (30.8) | 41 (32.3) | 22 (43.1) | 19 (25.0) |
